# Adenoid Cystic Carcinoma of the Bartholin's Gland: A Diagnostic Dilemma

**DOI:** 10.1155/2019/1784949

**Published:** 2019-08-18

**Authors:** Charmaine C. W. Lo, Jerome B. Y. Leow, K. Naing, Ken Jaaback, Thanuja Thachil

**Affiliations:** ^1^Faculty of Health and Medicine, University of Newcastle, Newcastle, New South Wales, Australia; ^2^Central Coast Cancer Centre, Gosford District Hospital, Gosford, New South Wales, Australia; ^3^John Hunter Hospital, Newcastle, New South Wales, Australia

## Abstract

Adenoid cystic carcinomas of the Bartholin's gland are extremely rare and are often misdiagnosed. There are currently no definite treatment guidelines. This article describes the case of a 33-year-old female who was managed at our centre for adenoid cystic carcinoma of the Bartholin's gland. She presented with a prolonged history of a vulvar lesion which was eventually diagnosed as adenoid cystic carcinoma of the Bartholin's gland. She was subsequently treated with wide local excision of the primary and inguinal lymph node dissection followed by adjuvant radiotherapy and chemotherapy. She had gross perineural invasion on MRI imaging. The present case highlights the diagnostic dilemma in this extremely rare cancer and the literature further explores the natural history and treatment options.

## 1. Introduction

Primary Bartholin's gland cancer (BGC) is rare and consists of 0.001% of all female genital cancers [1]. The variety of subtypes for BGCs include squamous cell, adenoid, epitheloid-myoepithelial, neuroendocrine, Merkel cell, lymphoepithelium-like, transitional cell, and adenoid cystic carcinoma [2]. Adenoid cystic carcinomas usually arise from minor and major salivary glands [3]. The other sites of origin include the nasopharynx [4], lacrimal glands [5], skin [6], trachea [7], breast [8], and vulva [9]. As of 2018 only 350 cases of the adenoid cystic subtype have been reported in the Bartholin's gland [10]. BGCs are often misdiagnosed because they are so rare but also often present as a mimic of a benign Bartholin's gland pathology. There are currently no clinical guidelines for the treatment of BGCs. Below, we describe a case of the adenoid cystic subtype of a BGC seen at our center. The aim of the present study is to highlight the importance of early diagnosis of this rare cancer and to explore the treatment options in order to improve the prognosis. Our case interestingly showed pudendal nerve invasion which could have been missed if the natural history was not well explored.

## 2. Case Presentation

A 33-year-old, ECOG 0 female was referred to our centre for opinion and management of postoperative locally invasive adenoid cystic carcinoma of the Bartholin's gland (ACCBG). She was nulliparous on an oral contraceptive, with menarche at the age of 15. Gynaecological and family history was otherwise unremarkable.

**Figure 1:**
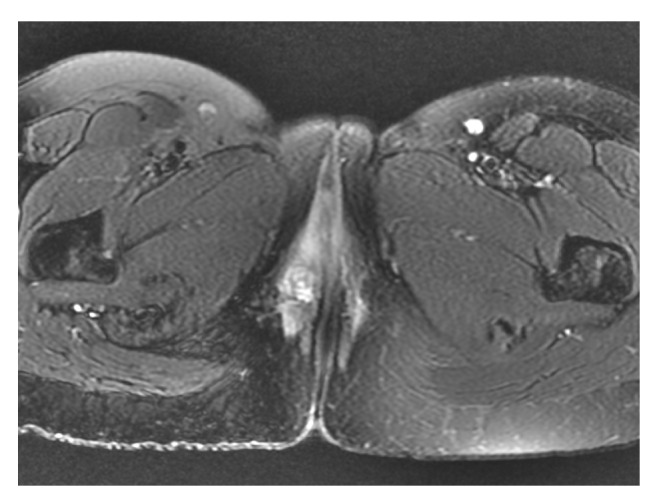
Preoperative T2 axial MRI though the pelvis demonstrating ACCBG lesion in the right vulva.

She first presented to a hospital in 2009 for a right vulvar lesion. Biopsy of this lesion was benign, and the lesion resolved without intervention. In 2013, the patient noticed a new right perineal lesion. CT, MRI, and bone scans were organized by her gynaecologist. Investigations were unremarkable except for a lesion in the ischial tuberosity on MRI. The lesion was deemed benign after review by orthopedic surgeons. 4 years later, the patient began to experience dyspareunia associated with a burning sensation. An MRI performed in June 2018 showed soft tissue swelling in the perineal region and a 14x13x13mm rounded soft tissue mass at the right posterolateral margin on the vaginal introitus consistent with a Bartholin's cyst. The lesion appeared to infiltrate the vaginal wall in the anteromedial margin, but this did not cross the midline (Figure 1).

**Figure 2:**
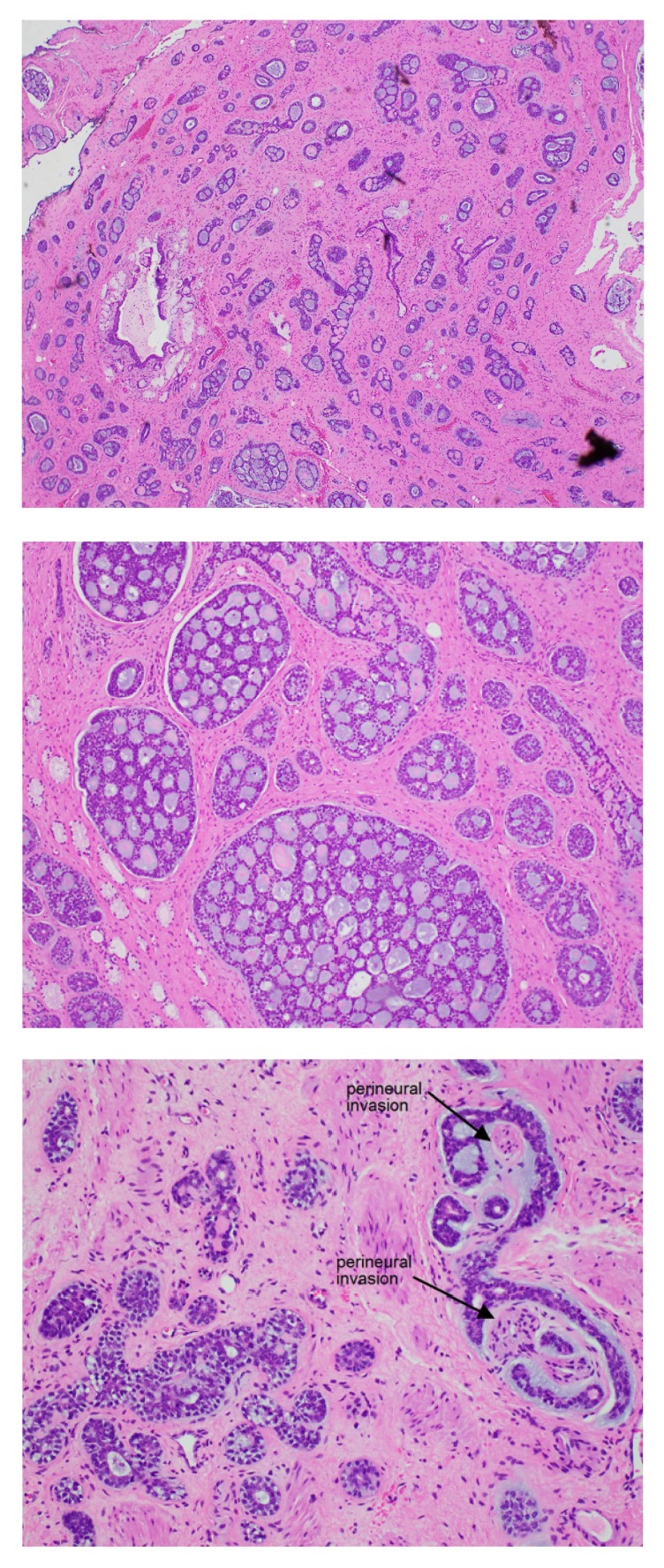
Histological slide of the ACCBG specimen. Cribriform and tubular glands infiltrating the submucosa of the vulva. There are pseudoglandular spaces with excess basement membrane material and mucin. Foci of perineural invasion are present. The tumour involves excision margins.

A biopsy was performed with histological features consistent with adenoid cystic carcinoma followed by wide local excision of the right vulva. The specimen spanned 43x25x32mm, and the tumour involved the excision margins. On histology, the specimen had foci of perineural invasion and invaded fibrous tissue, fat, and skeletal muscles (Figure 2). One month later, 8 lymph nodes were dissected, none of which were positive.

**Figure 3:**
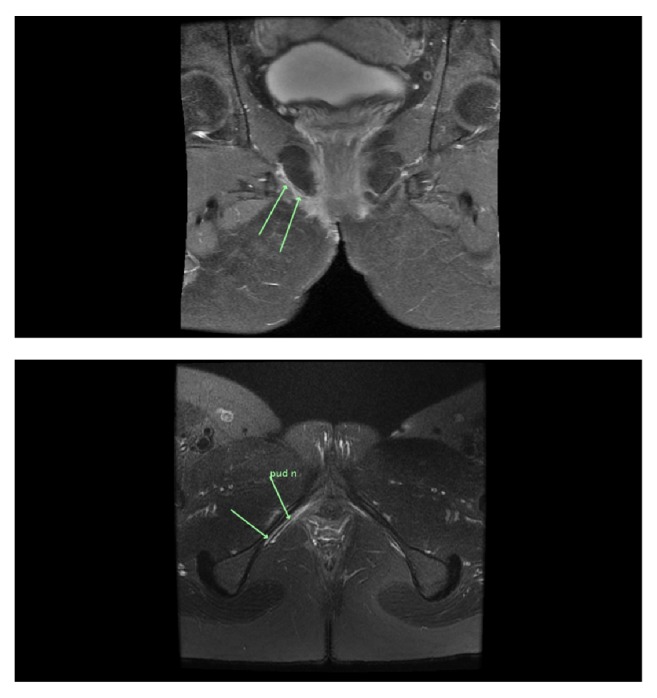
Postoperative T2 axial MRI through the pelvis demonstrating high T2 signal in the right pudendal nerve.

PET scan 2 months after surgery showed FDG uptake consistent with postsurgical changes and uptake in a right axillary node that was likely inflammatory rather than a distant metastasis. Further MRI contrast scans to assess extent of perineural invasion showed linear enhancement along the course of the perineal branch of the right pudendal nerve, terminating before Alcock's canal (Figure 3).

The consensus of the Gynaecologic Oncology Multidisciplinary Tumour Board was to proceed with adjuvant chemoradiation therapy. The patient received radiotherapy postoperatively to the tumour bed to a total dose of 66 Gy and to the right pudendal nerve to 59.4 Gy in 33 fractions by a VMAT technique with concurrent weekly 40 mg/m^2^ cisplatin chemotherapy which she tolerated well. The ovaries were spared to prevent premature ovarian dysfunction. At her 3 month posttreatment follow-up, there was clinically and radiologically no evidence of locoregional recurrence. The patient is planned for close surveillance.

## 3. Discussion

### 3.1. Etiology and Disease Progression

The etiology of ACCBG is unknown, though a series of ACCBG cases by Copeland has suggested that pregnancy may be an independent risk factor [11]. ACCBGs can present as both painless and painful masses [10, 12] and are usually solitary and unilateral. Clinically, they are associated with pruritus and a burning sensation, which reflects its nature to invade the perineum but has also presented with bleeding, dyspareunia, and discharge from abscesses [13]. They usually occur in women between 40 and 60 years old, with an age range of 29 to 76 years [7].

Histologically, most ACCBG occurs in a ‘cribriform' pattern [14] and is diagnosed using the haematoxylin and eosin stain [15]. As described by Copeland [11], the ‘cribriform' pattern is characterized by anastomosing cords of cells surrounded by acellular spaces containing mucin and hyaline. Tumour cells are small and basaloid [1], with scant cytoplasm and normal nuclei. They usually have extensive perineural and skeletal muscle invasion [1, 11, 16]. ACCBGCs are distinct from other subtypes of BGCs and seem to arise from myoepithelial cells of the Bartholin's gland [17].

ACCBG's are slow growing but are locally aggressive and have a high recurrence rate [18]. Usually several local recurrences precede distant metastasis. Distant metastatic sites include the lungs [18] and less commonly brain [12] and bone [16]. Patients with ACCBG treated with radical local excisions can have 5, 10, and 15 year progression-free rates of 47%, 38%, and 13% respectively [11]. Overall survival rates at 5, 10, and 15 years are 71%, 50%, and 51% as reported by Copeland et al. [11].

### 3.2. Management of ACCBGs

There are no standard treatment guidelines for these extremely rare tumours. Surgery is currently the mainstay of treatment. Surgical options reported include wide local excisions, hemivulvectomy, simple vulvectomy, and radical vulvectomy with and without inguinal and/or femoral lymphadenectomy [11, 16, 19, 20]. There is no consensus as to the best surgical approach. While some advocate for radical vulvectomy [13, 21] as it results in lower rates of positive margins, others advocate for a more conservative surgical approach to reduce delay to chemoradiation as ACCBGs have early local dissemination and margin status does not affect recurrence rates [13]. However, up to 8% of BGCs cannot be treated surgically [22].

Adjuvant radiation is also used to treat ACCBGs with a range of doses though there is no consensus regarding the role, extent, or total dose of radiation [23]. It is recommended especially in patients with positive margins on resection [24, 25]. Previous case reports [12, 26–28] have reported radiotherapy doses ranging from 50.4 Gy to 66 Gy [27]. Extrapolating from Head and Neck adenoid cystic carcinoma series, it is worthwhile to rule out gross perineural invasion and to treat the entire grossly involved nerve [3, 29–31]. Clinicians should discuss fertility options with premenopausal patients prior to adjuvant radiotherapy.

There has been one report which stated a similar overall survival with primary radiation or chemoradiation compared to other surgical series [32], but to date this is not the preferred approach.

There is less evidence for chemotherapy in ACCBG compared to adjuvant radiotherapy. A variety of chemotherapy regimens used include drugs such as paclitaxel, cyclophosphamide, cisplatin, methotrexate, doxorubicin, and 5-fluorouracil [10, 33, 34]. Chemotherapy regimens are usually used in conjunction with radiotherapy or after recurrence of ACCBGs after surgery and adjuvant radiotherapy treatment [10, 34]. Immunotherapy has also been used with anecdotal reports of excellent response [33].

## 4. Conclusion

Adenoid cystic carcinoma of the Bartholin's gland is an extremely rare tumour with no established diagnostic or therapeutic pathway. We present a case of a 33-year-old woman with adenoid cystic carcinoma of the Bartholin's gland treated with surgery followed by adjuvant concurrent chemoradiotherapy, preserving ovarian function. Our case interestingly showed invasion of the pudendal nerve and the patient in fact had prolonged signs and symptoms for a few years before the eventual discovery of adenoid cystic carcinoma. This rare tumour often poses substantial diagnostic and therapeutic dilemmas. Case reports and multi-institutional pooled studies are imperative in contributing to future diagnostic and treatment guidelines of this extremely rare malignancy.
